# An Introductory Overview of Open-Source and Commercial Software Options for the Analysis of Forensic Sequencing Data

**DOI:** 10.3390/genes12111739

**Published:** 2021-10-29

**Authors:** Tunde I. Huszar, Katherine B. Gettings, Peter M. Vallone

**Affiliations:** National Institute of Standards and Technology (NIST), Gaithersburg, MD 20899, USA; katherine.gettings@nist.gov (K.B.G.); peter.vallone@nist.gov (P.M.V.)

**Keywords:** massively parallel sequencing (MPS), next-generation sequencing (NGS), short tandem repeat (STR), sequence analysis, software

## Abstract

The top challenges of adopting new methods to forensic DNA analysis in routine laboratories are often the capital investment and the expertise required to implement and validate such methods locally. In the case of next-generation sequencing, in the last decade, several specifically forensic commercial options became available, offering reliable and validated solutions. Despite this, the readily available expertise to analyze, interpret and understand such data is still perceived to be lagging behind. This review gives an introductory overview for the forensic scientists who are at the beginning of their journey with implementing next-generation sequencing locally and because most in the field do not have a bioinformatics background may find it difficult to navigate the new terms and analysis options available. The currently available open-source and commercial software for forensic sequencing data analysis are summarized here to provide an accessible starting point for those fairly new to the forensic application of massively parallel sequencing.

## 1. Introduction

Next-generation sequencing (NGS) technologies transformed the field of genetics in the past decade. Descriptively referred to also as massively parallel sequencing (MPS), this high-throughput genomics method developed on various platforms provides genome-scale insights from data for the fields of medical diagnostics [1], epidemiology [2], population genetics [3], and more recently for forensic genetics [4,5,6,7] as well. The generation of massive datasets creates new challenges in data storage and security, analysis, interpretation, and comparable reporting, which is required to be consistent with traditional forensic genetics standards.

The field of forensic genetics often requires its scientists to have widespread knowledge in related fields such as general genetics, chemistry, physics, physiology, and pathology; however, bioinformatics was rarely among the skills in demand previously. With the introduction of MPS to the field came the generation of a greater amount of data. Due to the lack of readily available user-friendly software, such scarce skills became not just desirable but necessary for early adopters. In the beginning, software to interpret the sequencing data was only developed by research laboratories, naturally with none of the usual emphasis on an attractive graphical user interface (GUI) but focused on functionality and required the users to comfortably navigate the command line. Most analysts working in the forensic DNA laboratories are familiar with running software on the Windows operating system; even those using their Macintosh with the Unix-based operating system rarely would open their terminals and engage in command line operations. Suddenly, the need for data analysis required skills in navigating a whole new world of software running on Unix- and Linux-based computers, and while purchasing such computers was simple enough, gaining the skills to use the software may seem more challenging [8]. Such limitations were recognized and, with time, more software options were developed from research laboratories for the needs of the forensic community, some even offering versions run on Windows or more accessible web-based software. To date, several commercial options also entered the arena, offering to close the gap by providing visual, easy to use and ready-to-export solutions, which would satisfy those in need of quick answers and no particular desire to look ‘under the hood’.

Some forensic laboratories already established analysis of the mitochondrial DNA (mtDNA) using Sanger sequencing, and for those laboratories the introduction of MPS brings benefits mostly from the upscaling of the sequencing processes, lowering costs and manual workload, speeding up and automating the analyses. Furthermore, MPS of mtDNA may allow insight into more nuanced phenomena, such as low-level heteroplasmy, length heteroplasmy, and better detection of low-level mixtures. Short tandem repeat (STR) typing, however, had never used sequencing as a standard for forensic analysis, therefore the analysis of this new type of data introduces challenges. DNA analysts are familiar with interpreting STR data from capillary electrophoresis (CE) electropherograms from the last two decades, and many of the CE features are transferable to sequencing, e.g., the length-based allele names, the electrophoretic peaks, and the stutter artifacts. The application of sequencing offers an extra dimension of information for the markers, which drives the ongoing efforts to standardize the nomenclature of the sequence-level data, with the requirement to be back-compatible with the length-based allele names. Software solutions developed individual reporting formats that are sometimes difficult to reliably compare; however, most of these also provide a visual representation of the data, comparable to the already familiar electropherograms, and detailed counts of coverage read depth, similar to CE relative fluorescence unit (RFU) values. Despite the variable formats, these efforts aim to provide a human-readable sequence structure, as well as a sequence string format for universal comparison of the detected sequence variants. One area of non-consensus is the degree or range of reporting of the flanking regions surrounding the markers. While this is mostly influenced by the chemistry used, interpretation of these regions may be optional, dependent on settings, or may even be omitted; therefore becoming a potential source of discrepancy between analysis methods. Similar to reporting from CE data, the analysts will be required to report which kit they used, supplemented with the genomic range of reporting to avoid such discrepancies. While adjustments to reporting will become straightforward with nomenclature standardization and the available software options are increasingly user-friendly, the most critical adaptation for the analysis of STR sequencing data is reaching a comfort level with this data type, developing some basic bioinformatic skills to process data and interpret sequence variants routinely or in challenging cases.

Here we provide a short compendium of the various software and algorithm options available for sequencing data analysis to date with a focus on the forensic context. We aim to provide an accessible guide for forensic professionals starting to implement these novel sequencing methods into their standard forensic DNA analysis workflows.

## 2. Rationale of Massively Parallel Sequencing Data Analysis Methods for STRs

**Figure 1:**
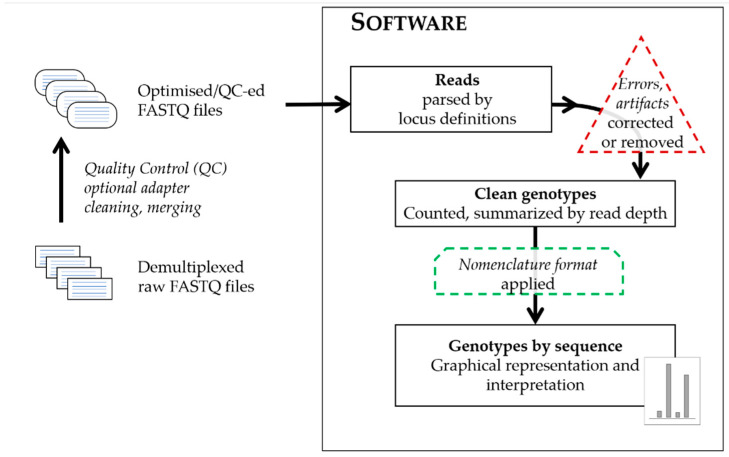
Schematic representation of general forensic MPS data processing steps.

True to the proverbial concept of bioinformatics, that ‘there is more than one way to solve a problem’, individual algorithms indeed differ, but regardless of which programming language they use, on which operating systems they run or which sequencing data type, or platform they can process, the general approach is broadly similar and summarized on the schematic graph in Figure 1.

The input files are text files containing sequence data in different formats generated by the sequencing platforms: files of sequence data with or without quality values for each base call in each read (FASTQ or FASTA), or sequence alignment files and their indices (BAM and BAI). The sequencing reads from the input files are parsed by using a defined set of attributes with characteristics of the targeted markers by which to filter. The terminology of the software describing these attributes significantly differ, therefore Table 1 compares not just the software themselves, but the verbiage for the files providing locus definitions and names for the landmarks of the targeted loci. These files provide configurations for the analyses in respect to the range and specificity of sequence targeted, by allowing strict or flexible matching to the short sequences landmarking the targeted loci and their immediate flanking regions. These landmark sequences anchor the reads to the selected loci, and often coincide with known or presumed primer regions of the amplicons. The targeted markers are also described by their repeat motifs and/or structure, which increases the locus-specificity and allows for the precise recognition of allele variants. Approaches differ as to whether software only recognizes a predefined set of allele variants aligning reads to these references, or could recognize and call undefined, novel variants, and furthermore, capable of creating various possible combinations of expected alleles just from the provided repeat blocks of the array. Regardless of the approach, the reads of each marker are tallied and summarized in the form of a read depth value (or coverage) for each allele. The recognition of a group of reads as alleles are also facilitated by adjustable analytical thresholds separating signal from noise. The relationship between observed sequences is often used to categorize calls as true alleles or their derivatives (stutter or reads with errors). Some software offers options to flag, remove, and/or correct potential artifacts and errors from sequencing. At the end of the process, allele calls are designated based on adequate coverage surpassing thresholds for interpretation and being excluded as artifacts. The common denominator of any software approach is the generation of sequence strings as the ultimate comparable form of sequence alleles, a requirement [9,10] for publishing population study sequence variants that allow for concordance checking between methods; with the caveat of different analysis ranges may still generate discrepancies between different methods. While such sequence strings are easily comparable by computer programs, this is not true for human analysts, therefore the software also reports a human-friendly format of the sequence alleles in their preferred nomenclature. These usually are presented in a ‘bracketed’ form with the counts of the repeat blocks summarized using brackets (e.g., [GATA]8); furthermore, these formats could address the marker, genomic location analyzed, the length equivalent of the allele and may also include any flanking region variation observed when compared to the human reference genome (usually the most recent version, GRCh38). Most software, apart from the standard outputs of the sequence strings, read depths and a form of bracketed nomenclature, also provide a visual output: a graphical representation of the detected alleles in a familiar histogram format, which being similar to the electropherogram peaks aids the transition of analysts from STR typing by CE to sequencing.

A new phenomenon introduced by using bioinformatic software for forensic DNA analysis is the occasional appearance of bioinformatic null alleles. These are the bioinformatic equivalents of null alleles in CE where sequence variation underneath the primer binding sites could impair or prevent amplification of the actual alleles. In the case of bioinformatic nulls, the amplification is not compromised and the sequencing reads are present in the raw data files, but there is an unexpected sequence variation underneath the landmark regions of a locus that a software uses to recognize locus-specific reads. While most software allows for ‘wobble’ or approximate matching in these landmark regions, this sequence variation can be significant enough for the software to fail to recognize and analyze the true reads in the filtering process, thus resulting in a null allele. The best prevention of bioinformatic nulls being reported in profiles is the use of a secondary data analysis method, which can be particularly useful in forensic validations or population studies. Using a sequencing platform-specific software in combination with another commercial, free or open-source software can largely eliminate the chance of bioinformatic nulls remaining unrecognized. In the case of the custom loci set developed in-house, where only open-source software can be used, it is good practice to use multiple software to call alleles, or at least use the same software with different settings in their locus definition files specifying different landmark regions, to avoid the occasional bioinformatic nulls.

Analysis of sequencing data requires access to adequate storage and safeguarding of the generated data. Local protocols need to be developed for the long-term maintenance and expansion of these resources, considering the size of the data files is not comparable to those originating from CE, often measured in gigabytes per run. Most of the following tools can be run on a standard laptop or desktop, but high-performance computing resources can be beneficial when processing a lot of samples.

## 3. Freely Available Software

In the early stages of the application of massively parallel sequencing to the forensic field, most solutions were developed in academic settings as a necessary research tool to be able to characterize and analyze data generated by sequencing platforms [11,16,20,22]. These approaches often focused on STR markers, occasionally offering options to analyze mtDNA data as well [18]. These software are freely available but assume the users have a basic level of bioinformatics skills allowing them to navigate and operate through the command line. Such basic skills can be obtained either through professional training [32] or self-taught courses via one of the several available online tutorials on ‘how to Linux’ [33]. Once the basic command-line skills are comfortably obtained, the following software can be run just as confidently through a terminal window as clicking an icon in a GUI. While there are a few software options available through web-based interfaces [23,24,25], some developers offer [21,34] or transition to [35] providing a version of their software that can be downloaded as a ‘Docker image’. This is a ‘ready to use’ packet of the program and all its dependencies required to run the application successfully, regardless of the underlying resources available locally [36]. A program, the Docker engine, facilitates the use of such packets on both Linux and Windows-based applications. While similar to virtual machines, this solution is more flexible and portable, as the isolated environment does not require a part of the hardware to be closed off, but rather creates such containers on a software level. This form of software availability improves not only data security, satisfying those who cannot allow data file exchange outside of their local laboratory but can also make these applications more accessible for those who are just beginning their journey with software operated through the command line.

### 3.1. STRait Razor

This software, designed to analyze reads from sequenced amplicons targeting STRs, was first published in 2013. Its evolution went through iterations from STRait Razor [11], v2.0 [12], v2s [13], v3.0 [14], and STRait Razor Online (SRO) [15] improving its processing speeds and output files, extending its analysis to the flanking regions and providing secondary analysis tools, such as additional workbooks for visual interpretation of the data using histograms and reporting sequence alleles following the International Society for Forensic Genetics (ISFG) early considerations [37].

The software uses FASTQ files as an input and versions prior to SRO required command line navigation. Detailed help files and guides are available, instructing users how to run an analysis by entering a command with the desired options. The file that sets the locus definitions is referred to as the ‘config file’ and the landmarks on each side of the loci are called the ‘anchors’. These modifiable config files are included for the currently available main sequencing kits, or custom files can be generated by the user. The output are simple text files, which can be processed further either by the provided Excel workbooks, the online platform, or custom scripts for the advanced users. These additional processes can summarize the results in a tabular and a visual format and facilitate additional insights such as allele nomenclature, stutter analysis, or sequencing error profiles. While previous versions could be run on the command line (using a Mac or a Linux computer), the v3.0 of this software can also run using the Command Prompt in Windows. The latest version (SRO) introduces the main functionality of the software in an online tool format, suitable for quick analysis of individual files, without the use of a Unix or Linux environment. The online format significantly decreases the need for bioinformatics skills; however, for batch processing a large number of files or for the use of custom settings running the downloaded command-line version of SRO is more practical.

The software includes config files for the commercially available sequencing kits and a default set of predefined alleles to call theses from the sequencing reads it analyzes, therefore any undefined sequence allele by default would require the user to establish an appropriate nomenclature. In such cases, the software may label the unrecognized variant as ‘novel’ by default, however, the variant may have been reported in more up-to-date literature or increasingly available databases [38,39,40].

The software is a general starting point for those interested in exploring their data further, specifically to be able to provide an unrestricted reporting of the flanking region variants [41]. It has proven useful in providing a secondary analysis to commercial software outputs as a means for eliminating bioinformatic null alleles [42].

### 3.2. FDSTools

This software is also designed to analyze reads from amplified STRs, with later versions offering the capability to analyze mtDNA results [43] from sequencing data. The evolution of the software through its iterations starts with the standalone TSSV tool [16] recognizing repetitive motifs in the reads, which was integrated into the FDSTools bundles v1.0, later v1.1.1 [17,18]. The latest version (v2.0) was expanded by an integrated nomenclature package STRNaming [19]. The software is a bundle of several tools to be used in the analyses of sequencing data from raw FASTQ files. Analyzed loci and their analysis attributes are defined by a ‘library file’, including their landmark regions referred to as ‘flanks’. Results include coverage values with options for different outputs including bracketed and string formats. The package includes several additional tools for stutter analysis and correction, databasing, and visualizations as well. The addition of the STRNaming module eliminates the need for user input on the locus definition files. Instead, the program now automatically recognizes repetitive sequences in the reference sequence using these as the preset preferences for bracketing interpretation of the sequence reads and, as such, automates nomenclature classification of the called alleles. The addition of this module facilitates the ongoing efforts to reach a unified nomenclature for the standard human forensic STR markers [37,44,45].

The software is a good starting point as a secondary analysis option with additional flexibility for those interested in building custom solutions for their more specific needs beyond standard reports [17,46,47]. The offered modular tools and customization are ideal for stutter analysis or the visualization of stutter restoration to the respective parent allele [48]. Those who appreciate graphics generated in a report-ready format will find the graphical HTML outputs useful [17]. Beyond the standard or custom niche sets of STR markers of human forensics, the software is an ideal tool for those developing wildlife forensic markers with the need for flexible software adaptable to species identification from novel STR multiplexes [49].

### 3.3. STRinNGS

The software STRinNGS v1.0 [20] was one of the early approaches available on request developed by researchers. This tool required command-line skills to analyze the data and use the output files in further scripts to summarize stutter and error profiles observed. The recently released v2.0. [21] is openly available to download for local use and has been updated to provide a more refined set of criteria for improved reliability in allele calling including error filtering, identifying stutter reads, and flagging unusual sequences for manual review. STRinNGS accepts FASTQ files as input and runs the settings via its locus definition file which is referred to as the ‘configuration file’ where it defines the marker landmarks as ‘flanking sequences’. To accommodate the need for quality control (QC), the software offers an output format that can be used directly for submission to STRidER [9]. This site (https://strider.online/) is dedicated to the QC of autosomal STR population data sets, providing unique identification numbers as proof of data passing their checks.

The software is a good alternative as a secondary analysis to eliminate bioinformatic nulls in the analysis and is now an improved tool that helps the analyst with the manual review by providing several optional flags and settings. The software reports a format in line with guidance from the forensic community [9,10,37] as well as its own developed format for allele nomenclature which is easily comparable with other free software outputs for concordance. It provides clear indications of the genomic locations, the length-based alleles, the sequence structures, and the flanking variations [50] and, for the convenience of the user, includes the sequence strings analyzed.

### 3.4. MyFLq

One of the earliest software solutions for forensic STR data analysis from MPS was developed [22] in a form of a web-based user-friendly application using FASTA or FASTQ files for input. In the past, this was also available as an integrated online tool on BaseSpace [23], for use with Illumina sequencing data output. For the practicality of analyzing sensitive data locally, a desktop version of the software is also available to download [22] or provided as a Docker-container file [34] to be downloaded as a functional package and run locally. To help recognize non-predefined true alleles, MyFLq can estimate whether an unrecognized allele is truly a novel allele or a result of errors. The landmarks defining the loci analyzed are referred to as ‘primers’, however, this does not necessarily mean that they completely overlap with the primers in the amplification reaction. The approach uses a dynamic calculation of the flanking regions and the region of interest (ROI), rather than a static definition of repeat region and flanking regions. The ROI, the variable part of the sequence, is compared to the reference alleles and allows an easy interpretation of SNPs as well as STR length polymorphisms within the analyzed region. The output is a report of the sequences with their sequence and the derived length alleles as well, including visualization of the results.

The use of this software can be interesting for those who want an alternative analysis when comparing methods and those who are interested in viewing their data in a simple, non-bracketed nomenclature format. MyFLq has the potential to work with SNP and mtDNA data as well. This approach could also be useful for working with new STRs or non-human STRs, capitalizing on the flexible approach of locus analysis which can adjust to a dynamically growing reference allele database.

### 3.5. ToaSTR

This software offers a user-friendly graphical web-based solution for the analysis of STR data from sequencing. It does not require bioinformatics expertise from the users as it provides an intuitive GUI to analyze data from FASTQ or FASTA files. Web-based software options often face questions about data security and laboratories may be restricted from uploading sensitive data to the web, therefore the developers currently provide access to this secure web-based tool upon request [24]. Those who require further assurance will welcome the recent update that will move the web-based application at the end of 2021 to a Docker-based format [35], allowing the software to be downloaded as a functional unit operating securely locally.

ToaSTR defines the analyzed loci together as a ‘panel’ and refers to the landmark sequences as ‘recognition elements’. The panels to be analyzed are customizable and therefore independent of the sequencing platforms and kits [51]. The software includes predictive stutter modeling allowing an automatic classification of the observed sequences and the differentiation of artifacts. The reporting nomenclature format of ToaSTR is aligned to the ISFG considerations [37] and includes graphical visualization of the results.

Until the introduction of the new format of the software, the web-based version can still prove to be useful for analyzing training data and experiments or mobile demonstrations that require quick, visually appealing outputs.

### 3.6. Altius

Altius was developed as an independent secure Cloud-based software optimized for high-throughput data processing from FASTQ files. As users access this intuitive GUI through a web browser, it requires no bioinformatic expertise. The software is capable of processing MPS data of a predefined set of STRs (autosomal, X- and Y-STRs) [52,53] generated by different platforms, including the MinION. The analysis is robust and is ready to accommodate batch data processing [25]. The target regions for locus identification parameters are adapted from STRaitRazor v2.0 [12] and locus definitions are collated in a lookup table for the software to identify the targeted loci. The results are output to a MySQL database and exportable reports are provided for the sequences, including full sequence strings, a visual output of the results, and a format of nomenclature in line with the considerations of ISFG [37]. Data security for this software is provided by the resources of Amazon Web Services, allowing users to set their locally required level of access-control measures. Because Altius is using the secure cloud system, access is provided upon request and after authentication.

## 4. Commercial Software

Apart from the freely available academic software, there are several options offered by commercial companies. These are either provided as a supplement to the vendors’ own sequencing chemistry and platform or developed as standalone solutions for analyzing raw data output from various sequencers.

In general, these are user-friendly programs with visually appealing graphical interfaces and with limited options to customize processes, all designed to provide a streamlined process of hassle-free analysis, familiar graphical output, and presentation-ready results. Many of these offer options for mtDNA analysis as well as STR data analysis, both generated on the sequencing platforms. Commercial software is designed to make the introduction of MPS easier to any new user, building confidence working with sequence data; however, there is less control of the algorithms and occasional troubleshooting requires the assistance of the companies. For high-throughput routine laboratories, these qualities are attractive and the reliable convenience offered by these programs could justify the cost.

### 4.1. GeneMarker HTS (SoftGenetics, State Collage, PA)

GeneMarker HTS [27] offers an integrated solution for analyzing sequencing data from mtDNA, STRs, and SNPs generated on either Illumina or Ion Torrent platforms. The software is validated for mtDNA data analysis [54,55]. It can be used to analyze the mtDNA control region or the whole mtDNA genome, as required [56]. The STR analysis utilizes an in-built panel for the Promega PowerSeq 46GY kit (Promega, Madison, WI, USA), using FASTQ files generated from an Illumina MiSeq (Illumina, San Diego, CA, USA), alternatively, a panel for custom chemistries can be used for analyzing data from other kits. MtDNA and STR analysis (including flanking region variations) can be performed individually or simultaneously. GeneMarker HTS reports the length-equivalent of the sequence alleles, provides sequence strings and a visual interpretation of the results using histograms. An audit trail of changes and analysis settings are logged, and user access rights are controlled by its database. Demo and trial versions, training materials, and product support are available. GeneMarker HTS operates under Windows.

### 4.2. ExactID (Battelle, Colombus, OH)

Battelle’s ExactID [26] offers another fully integrated agnostic software solution designed for professional use in government agencies and crime laboratories. The sequencing platform-independent software analyzes data from FASTQ files generated from various chemistries targeting forensic markers, such as autosomal, X- and Y-STRs [57,58], SNPs, microhaplotypes, and mtDNA. The analysis settings are defined in ‘config files’ for the various marker panels. The user-friendly GUI offers a familiar display of the observed alleles in a histogram format along with the length-equivalent alleles and the bracketed sequence alleles in line with the ISFG considerations [37] for STRs. The software can recognize previously undefined alleles and to report flanking region variation. The results can be exported in multiple formats: .pdf file with tabular and graphical summaries, .csv files for further external analysis, and an additional .sef file format for evidence preservation. Furthermore, ExactID offers additional intelligence leads by interpreting data relating to phenotypic markers and biogeographical ancestry using the Battelle Avatar plugin. Audit trail and user access control are provided by the software. ExactID operates under Windows.

### 4.3. MixtureAce (NicheVision, Akron, OH)

MixtureAce [28] the plugin tool for the ArmedXpert software offers a user-friendly option to analyze MPS data from FASTQ files for STR (autosomal, X- and Y-STR) markers [59,60] with the benefit of the integrated hash-based Sequence Identifier (SID) nomenclature [61], a unique abbreviated format of sequence-based alleles designed to identify the relationships between sequences. MixtureAce uses the SIDs to recognize reads of stutter or other predefined artifacts using customizable thresholds and thus facilitates the recognition of reads not filtered out as true alleles. Undefined artifacts still need to be manually curated [60]. The software reports within the ranges of sequences encompassing the STRs following the UAS flanking region report [44]. This ready-to-use solution can report from a single source or interpret mixed samples using another ArmedXpert plugin: Mixture Interpretation. MixtureAce operates under Windows.

### 4.4. CLC Genomics Workbench (QIAGEN, Hilden, Germany)

CLC Genomics Workbench [62] is a genomic bioinformatic tool collection developed by and offered from Qiagen for comprehensive sequencing data analysis in general. This tool allows customization of its collection with plugins, such as the AQME [29], the toolbox specifically developed in collaboration with AFDIL to accommodate forensic-specific mtDNA sequence analysis for data generated from any MPS platform. AQME also includes haplogroup estimation and phylogenetically consistent nomenclature to facilitate reporting of the results. This specific plugin can be applied within the CLC Workbench framework.

### 4.5. Universal Analysis Software (Verogen, San Diego, CA)

Universal Analysis Software (UAS) [30] is the custom software of the MiSeq FGx sequencing platform that can analyze sequencing data from forensic markers using specific modules for the ForenSeq line of kits. Currently available chemistries target STRs (autosomal, X- and Y-STRs), SNPs, and mtDNA. Raw data is directly processed from the sequencer to generate demultiplexed raw sequence output FASTQ files. This is then further analyzed within the software using alignment to the human reference sequence and variant calling from the sequences at the range reported by the software. To extend the reported range outside of the repeat region an additional flanking region report is also available in the form of an excel file. To further analyze variation outside of expected flanking region variations, the raw FASTQ files can also be exported and processed by external software for independent analysis and concordance. The software is validated together with the platform and chemistry as the MiSeq Forensic Genomics System [63,64] supported by training and direct product support from the vendor.

The GUI is designed to be intuitive and user-friendly and with default and additional modules for different forensic genomic applications for the FGx platform, such as the STR analysis module or the data analysis for mtDNA sequencing chemistries. A supplementary analysis generates investigative leads, such as the estimation of phenotypic markers (hair and eye color) and biogeographical ancestry estimation of the samples [65,66]. Furthermore, genomic applications can analyze data generated from dedicated SNP panels for SNP-based identification of degraded remains; or can pre-format the generated data for downstream use in databases specific to the application of forensic genetic genealogy (FGG). FGG is an investigative tool for identifying distant kinship of a sample using databases built from ‘direct-to-consumer’ (DTC) genealogy DNA test results, data volunteered by citizen scientists. The generated data is formatted to be comparable with the markers in the database allowing to facilitate the investigation of serious crimes or to identify unidentified human remains [67].

### 4.6. Converge Forensic Analysis Software (Thermo Fisher, Waltham, MA)

Converge Forensic Analysis Software [31] is the comprehensive validated software customized to the HID Ion S5 sequencing platforms of Thermo Fisher. Converge is designed for this specific sequencing platform and visualizes the analyzed results obtained from the Torrent Server via the HID Genotyper plugin. It has modules specific to workflows of the offered chemistries targeting specific forensic markers: STRs including multiple markers for sex-determination [68], mtDNA control region, or the full mitochondrial genome [69]. Additional modules beyond STR analysis include those interpreting data from kits targeting selected SNP sets, which can establish identity from degraded samples [70,71] or can provide investigative leads and estimate biogeographic ancestry [72]. Data organization in Converge is optimized and streamlined around case management. The software and chemistries are validated for mtDNA analysis [73] and the users are supported by training and documentation from the vendor.

Via the HID Genotyper plugin, the generated sequencing reads are demultiplexed and aligned to the default reference sequence in regions specified by the BED file. The BED files are specific to the chemistries targeting different marker sets. Both the chosen reference and the BED files can be customized. The generated data can be downloaded as alignment files (BAM and BAI) or alternatively can also be generated as FASTQ files to download for independent analysis and concordance analysis.

The GUI is designed to be intuitive for the sequence-based data and follows the familiar look of the vendor’s CE-based software (GeneMapper ID-X, Thermo fisher, Waltham, MA, USA) and it can integrate and compare the two data types for casework, paternity, and kinship calculations. For markers that are not currently supported by the offered kits and the software (for example chemistry targeting multiple Y chromosomal markers), sequencing can be performed using a custom set of amplicons [74,75]. The generated raw data then can be downloaded and analyzed with the available independent software options.

## 5. Other Software Options for Whole Genome Sequencing (WGS) Data

Without exhausting the list there are other software options available [76,77], and many were designed to identify and analyze STR markers from genome-wide sequencing data without a forensic focus. STRs, in general, may be medically relevant or used as markers for population genetics, and specific software has also been designed to identify other relevant tandem repeats to facilitate medical diagnosis or genotype of these markers [78,79,80,81,82,83]. Recent reviews [84,85] also provided an overview of several alternative software that can generate STR profiles from whole-genome sequencing data [86,87,88,89,90,91,92,93,94].

While these may not be the immediate focus of forensic analysts mainly interested in reporting the sequencing data from the targeted amplification of markers specifically curated for forensic purposes, WGS data analysis methods could prove useful in exploring alternative approaches with already available data sources or in research projects.

## 6. Tips, Tricks, and More Tools

Despite the evolution of software solutions for forensic MPS data, occasionally data analysis can come to a halt if suspicious results are observed. This could be an unexpected null or supernumerary allele, unreasonably low coverage, or confusing sequence structure. In case of concern, there are always a few options to investigate the reason for discrepancies. For example, one can investigate the observed coverage values in relation to the expected inter-locus balance, which can indicate failure to detect an allele in heterozygotes interpreted falsely as homozygotes (bioinformatic null alleles). Any software can potentially generate bioinformatic null calls, i.e., the inability to recognize and report a specific variant. The best approach to confirm any unexpected instances is to use multiple software (or at least multiple settings) for the analysis and perform a concordance check-in between analysis methods.

In-built software of the sequencing platforms (UAS and Converge) can offer investigative leads using SNP data from some of their chemistries. Additionally, the user can harvest the relevant SNP data and independently verify certain phenotypic traits: eye and hair color using the constantly updated and freely available tools (https://hirisplex.erasmusmc.nl/) hosted at the Erasmus MC University. The website offers options for a manual or automated upload of the SNP genotype data to verify the prediction of these phenotypic traits using the established results from relevant studies (IrisPlex [95], HIrisPlex [96], HIRISPlex-S [97,98,99]).

Visualizing variants often helps to understand how some nucleotide changes create unusual sequence structures. A useful tool for visualization is the Integrative Genomics Viewer (IGV) [100], where alignment and variant calling files can be viewed manually compared to the reference sequence. If the consensus sequence of the reads is not obvious by manual revision another tool, VisCoSe, may be of interest that can calculate and compare consensus sequences of multiple datasets [101].

It is a good practice to perform independent Quality Control of the raw data prior to analysis, starting by monitoring the main characteristics of the dataset before and after any additional clean-up steps, which can be done, for example, using the FastQC program [102]. The additional steps of detailed adapter trimming using additional software (for example Trimmomatic [103], Cutadapt [104], seqtk [105]) or the merging of paired-end reads (using FLASH [106], BBMerge [107], CASPER [108]) may improve the analysis downstream. There are instances where using additional clean-up tools on raw data can improve the analysis. For example, removing erroneous reads and/or low-quality parts of reads specific to chemistry and platform can lead to unambiguous allele calls and can even improve retrieved coverage values for the dataset.

Available open datasets are a valuable resource for those not yet engaged in massively parallel sequencing but interested to learn more about data analysis ahead of establishing a workflow locally. One such source is the Forensic DNA Open Dataset, published by the NIST Applied Genetics Group [109] at https://doi.org/10.18434/M32157. Open datasets for WGS data are also available at the 1000 Genomes Project data portal, the International Genome Sample Resource (IGSR) [110,111,112] (https://www.internationalgenome.org/home), and the variants found in these projects can be viewed at the 1000 Genome Browsers hosted at NCBI [113] (https://www.ncbi.nlm.nih.gov/variation/tools/1000genomes/).

## 7. Summary

In this review, the aim was to provide a short, digestible overview of the currently available software options, acknowledging the challenges for the bioinformatically non-specialist reporting forensic professionals of this field. DNA analysts already familiar with the CE-based analysis and software, but inexperienced in high-throughput sequencing, or those planning to generate sequencing data in the future, would benefit from this review.

All the presented software options perform well and selecting one (or as suggested here: more) over others may be due to personal preference, financial limits or the compatibility to already available equipment. If routine forensic casework laboratories engage in exploring these various options, the DNA analysts will better understand the sequence-level variation of the forensic markers and the advantages of incorporating sequence data analysis into their workflows. An increased comfort level with basic bioinformatics is a key step to utilizing the new possibilities introduced by MPS to the field.

## Figures and Tables

**Table 1:** Summary of characteristics of software for the interpretation of MPS data of forensic markers.

Software	Versions	Author/Vendor	Year	Accessibility	Runs on	Locus Definition	Landmarks for Loci
STRait Razor	v1.0	Warshauer et al. [11]	2013	free	Unix/Linux	config file	‘anchor’
	v2.0	Warshauer et al. [12]	2015	free	Unix/Linux		
	v2s	King et al. [13]	2017	free	Unix/Linux		
	v3.0	Woerner et al. [14]	2017	free	all platforms		
	Online	King et al. [15]	2021	free	online/ all platforms		
FDSTools	TSSV	Anvar et al. [16]	2014	free	Unix/Linux	library file	‘flank’
	v1.0	van der Gaag et al. [17]	2016	free	Unix/Linux		
	v1.1.1	Hoogenboom et al. [18]	2017	free	Unix/Linux		
	v2.0	Hoogenboom et al. [19]	2021	free	all platforms	STRNaming	
STRinNGS	v1.0	Friis et al. [20]	2016	on request	Unix/Linux	configuration file	‘flanking sequences’
	v2.0	Jonck et al. [21]	2020	free	Unix/Linux		
MyFLq	v1.1	Van Neste et al. [22,23]	2014	free	online/Unix/Linux	panels	‘recognition elements’
toaSTR	v1.0	Ganschow et al. [24]	2018	free	online	allele database	‘primer’
Altius	Cloud	Bailey et al. [25]	2017	on request	online	lookup table	‘target regions’
ExactID	v2.0	Battelle [26]	2015	commercial	Windows	config file	default
GeneMarker HTS	v1.0	SoftGenetics [27]	2017	commercial	Windows	default	default
MixtureAce	v1.0	NicheVision [28]	2018	commercial	Windows	default	default
CLC Genomics Workbench	AQME	Sturk-Andreaggi et al. [29]	2017	commercial	all platforms	non STR	non STR
Universal Analysis Software	v2.3	Verogen [30]	2021	commercial	Windows	default	default
Converge Forensic Analysis Software	v2.2	Thermo Fisher [31]	2019	commercial	Windows	BED files	default

## Data Availability

Not applicable.
